# Diagnostic accuracy of anthropometric indices for discriminating elevated blood pressure in pediatric population: a systematic review and a meta-analysis

**DOI:** 10.1186/s12887-021-03062-8

**Published:** 2022-01-04

**Authors:** Jun-Min Tao, Wei Wei, Xiao-Yang Ma, Ying-Xiang Huo, Meng-Die Hu, Xiao-Feng Li, Xin Chen

**Affiliations:** 1 Department of Epidemiology, School of Public Health, Dalian Medical University, No. 9, West Section of Lvshun South Road, Lvshunkou District, Dalian City, 116044 Liaoning Provence China; 2 Department of Neurosurgery, Affiliated Dalian Municipal Central Hospital, Dalian Medical University, Dalian, 116033 China

**Keywords:** Body mass index, Waist circumference, Waist-to-height ratio, Elevated blood pressure, Children and adolescents

## Abstract

**Background:**

Childhood obesity is more likely to increase the chance of many adult health problems. Numerous studies have shown obese children to be more prone to elevated blood pressure (BP) and hypertension. It is important to identify an obesity anthropometric index with good discriminatory power for them in pediatric population.

**Methods:**

MEDLINE/PubMed, Web of Science, and Cochrane databases were retrieved comprehensively for eligible studies on childhood obesity and hypertension/elevated BP through June 2021. The systematic review and meta-analysis of studies used receiver operating characteristics (ROC) curves for evaluating the discriminatory power of body mass index (BMI), waist circumference (WC), and waist-to-height ratio (WHtR) in distinguishing children with elevated BP and hypertension.

**Results:**

21 cross-sectional studies involving 177,943 children and 3–19 years of age were included in our study. Meta-analysis showed that the pooled area under the reporting receiver-operating characteristic curves (AUC) and 95% confidence intervals (CIs) for BMI, WC, and WHtR to detect hypertension of boys were 0.68 (0.64, 0.72), 0.69 (0.64, 0.74), 0.67 (0.63, 0.71), for elevated BP, the pooled AUCs and 95% CIs were 0.67 (0.61, 0.73), 0.65 (0.58, 0.73), 0.65 (0.61, 0.71). The pooled AUCs and 95% CIs for BMI, WC and WHtR of predicting hypertension were 0.70 (0.66, 0.75), 0.69 (0.64, 0.75), 0.67 (0.63, 0.72) in girls, the pooled AUCs and 95% CIs of predicting elevated BP were 0.63 (0.61, 0.65), 0.62 (0.60, 0.65), 0.62 (0.60, 0.64) respectively. There was no anthropometric index was statistically superior in identifying hypertension and elevated BP, however, the accuracy of BMI predicting hypertension was significantly higher than elevated BP in girls (*P* < 0.05). The subgroup analysis for the comparison of BMI, WC and WHtR was performed, no significant difference in predicting hypertension and elevated BP in pediatric population.

**Conclusions:**

This systematic review showed that no anthropometric index was superior in identifying hypertension and elevated BP in pediatric population. While compared with predicting elevated BP, all the indicators showed superiority in predicting hypertension in children, the difference was especially obvious in girls. A better anthropometric index should be explored to predict children’s early blood pressure abnormalities.

## Background

Hypertension, as a vital cardiovascular risk factor, is estimated account for approximately 50% of the coronary heart disease burden and 67% of the cerebrovascular disease burden [1]. Hypertension is being increasingly reported in children and adolescents. Children with high blood pressure (BP) have early vascular aging and are more likely to progress to hypertension as adults [2–7]. Numerous studies have shown children with obesity to be more prone to hypertension and elevated BP [8]. Since the 1990s, the prevalence of children who are overweight and obese has increased dramatically [9]. Although the increased rate of childhood obesity in some developed countries has plateaued, the prevalence is still high [10]. Childhood obesity is more likely to lead to adulthood obesity, which can increase the chance of many adult health risk, such as heart disease, hypertension and type 2 diabetes [11]. To overcome this crucial health problem, it will be important to identify an anthropometric index with good discriminatory power that is simple to measure and interpret.

Currently, weight status can be assessed by several anthropometric indexes, such as body mass index (BMI), waist circumference (WC), and the waist-to-height ratio (WHtR); however, which index better predicts hypertension is unknown. A meta-analysis suggested that WC and the WHtR are not superior to BMI in identifying elevated BP in children, but only 9 articles were included in the previous study, of which only one Asian study was from India [12]. Therefore, the research results may have some limitations. The number of relevant studies has increased substantially. Therefore, we conducted a full robust systematic review to determine which anthropometric indices should be recommended for screening purposes.

## Methods

This systematic review was conducted according to the meta-analysis of observational studies in epidemiology (MOOSE) criteria [13].

### Literature search strategy

We comprehensively retrieved the MEDLINE/PubMed, Web of Science, and Cochrane databases for eligible studies involving childhood obesity and hypertension. The relevant studies were published until June 2021 were included. In addition, the key words used in the search included “body mass index or BMI,” “waist circumference or WC,” “waist-to-height ratio or WHtR,” “elevated blood pressure or hypertension,” “children,” and “adolescent.” All of the reference lists of included studies were also manually retrieved so that no studies were overlooked. The complete search strategy was described in supplemental Appendix 1. Two independent reviewers evaluated all relevant articles and disagreements were resolved by discussion.

### Study inclusion/exclusion criteria

- Studies was limited to cross-sectional design;
- Studies must focus on children or adolescents, any ethnic group, age < 19 years;
- Studies assessing the association between anthropometric indices and pediatric hypertension or elevated BP;
- BMI and WC or the WHtR, measured at least one metric;
- Studies reporting receiver-operating characteristic (ROC) curve analyses with the area under the ROC curve (AUC) provided.
- All anthropometric indices in the study must be measured twice at least with the mean value recorded;
- Non- original article, such as reviews and letters, were not considered;
- Studies that involved adults only or failed to provide AUC values were excluded;

### Main anthropometric indices and measures

WC was measured to the nearest 0.1 cm by a non-elastic flexible tape in the standing position. The tape was applied horizontally midway between the lowest rib margin and the iliac crest [14]. BMI was calculated based on the weight divided by height squared (kg/m^2^). The WHtR was calculated by dividing the WC (cm) by height (cm). The blood pressure was measured at least two times in each subject and the average value was obtained. The criterion of hypertension was according to the Fourth Report on the Diagnosis, Evaluation, and Treatment of High Blood Pressure in Children and Adolescents (NHBPEP) [15] and other local criteria of hypertension have also been adopted. Elevated BP was defined as the average systolic BP or diastolic BP ≥ 90th or ≥ 95th percentile for gender, age, and height.

### Data extraction and quality assessments

Two reviewers independently extracted the following study characteristics from the included studies using a standard data extraction form: Study, study year, country, region, study design, characteristics, BP measurement device, sample number, age, hypertension criterion and anthropometric indices. The AUC with a 95% confidence interval (CI) or standard error was extracted. The methodologic quality of included study was evaluated by the Quality Assessment of Diagnostic Accuracy Studies-2 (QUADAS-2) tool [16]. The quality of the evidence was rated by GRADE guidelines [17].

### Statistical analysis

ROC analysis is a widely-used method to examine the discrimination power of anthropometric indices, because of its powerful efficiency and clear interpretation [18–21]. The AUC prefers a comprehensive summary of test performance. Perfect tests have a mean AUC close to 1, whereas poor tests have an AUC close to 0.5. Data on the mean AUC with 95% CI and sample size for each study were inputted into a database. The quality assessment of included studies was carried out by Review Manager version 5.3 (Cochrane Collaboration, Oxford, England), meta-analysis was performed using STATA version 15.3 (Stata Corp., College Station, TX, USA). If an anthropometric index had more than one type of transformation in one study, the z-score was prior because the z-score is a standardized value. The heterogeneity of the studies was measured using the *I*^*2*^ statistic. The choice of the model depends on the heterogeneity test results and the hypothesis of the theoretical effect size. If the heterogeneity test was not statistically significant and the heterogeneity was so small that it can be ignored, which mean the *I*^*2*^ was ≤50% or the *P* value was ≥0.05 was identified for heterogeneity among studies, then the theoretical effect size can be considered to be fixed, and the fixed-effects model can be used; on the contrary, if the heterogeneity was large, which mean the *I*^*2*^ was > 50% or the *P* value was < 0.05 was identified for heterogeneity among studies, and the change of theoretical effect size was assumed normally distributed, the random-effects model should be selected.

To further analyze the differences between BMI, WC and WHtR, subgroup analysis was used separately for region, hypertension criterion, hypertension diagnostic basis and BP measurement device. The regions of studies were classified according to continent (Europe, America and Asia); the hypertension and elevated blood pressure criterion contained average systolic BP or diastolic BP ≥ 90th and average systolic BP or diastolic BP ≥ 95th percentile; diagnostic basis included NHBPEP and other local criteria of hypertension. BP measurement device was divided into mercury sphygmomanometer and automated BP monitor. Potential publication bias was determined by Begg’s test, and sensitivity analysis was also performed.

## Results

### Included studies

**Fig. 1:**
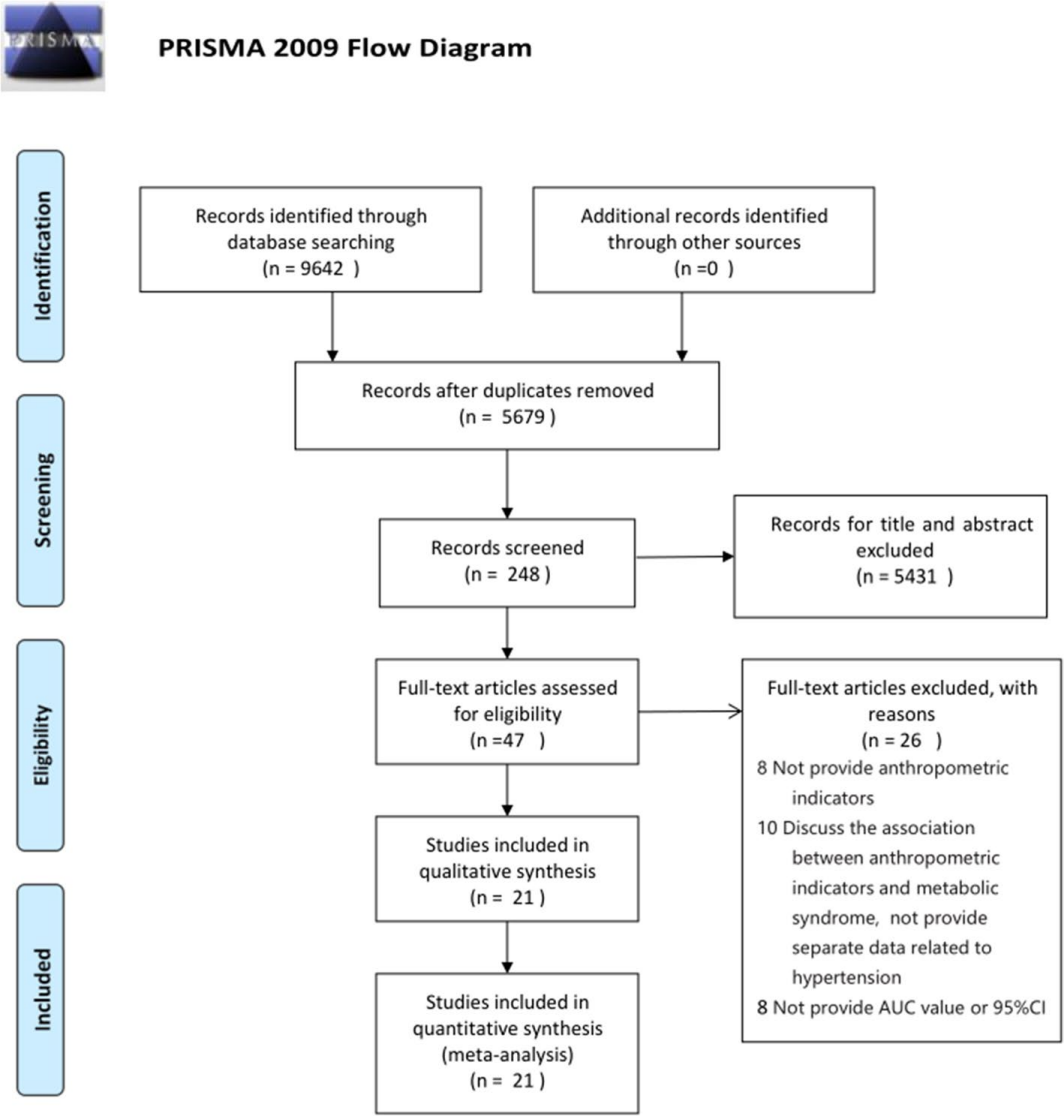
Flow diagram for study inclusion

The detailed study selection progress is shown in Fig. 1. Initially, 9642 articles were identified from the PubMed, Web of Science, and Cochrane databases, 26 studies were excluded after browsed the full-text, and 21 original articles were included for the meta-analysis in the end [6, 22–41].

**Fig. 2:**
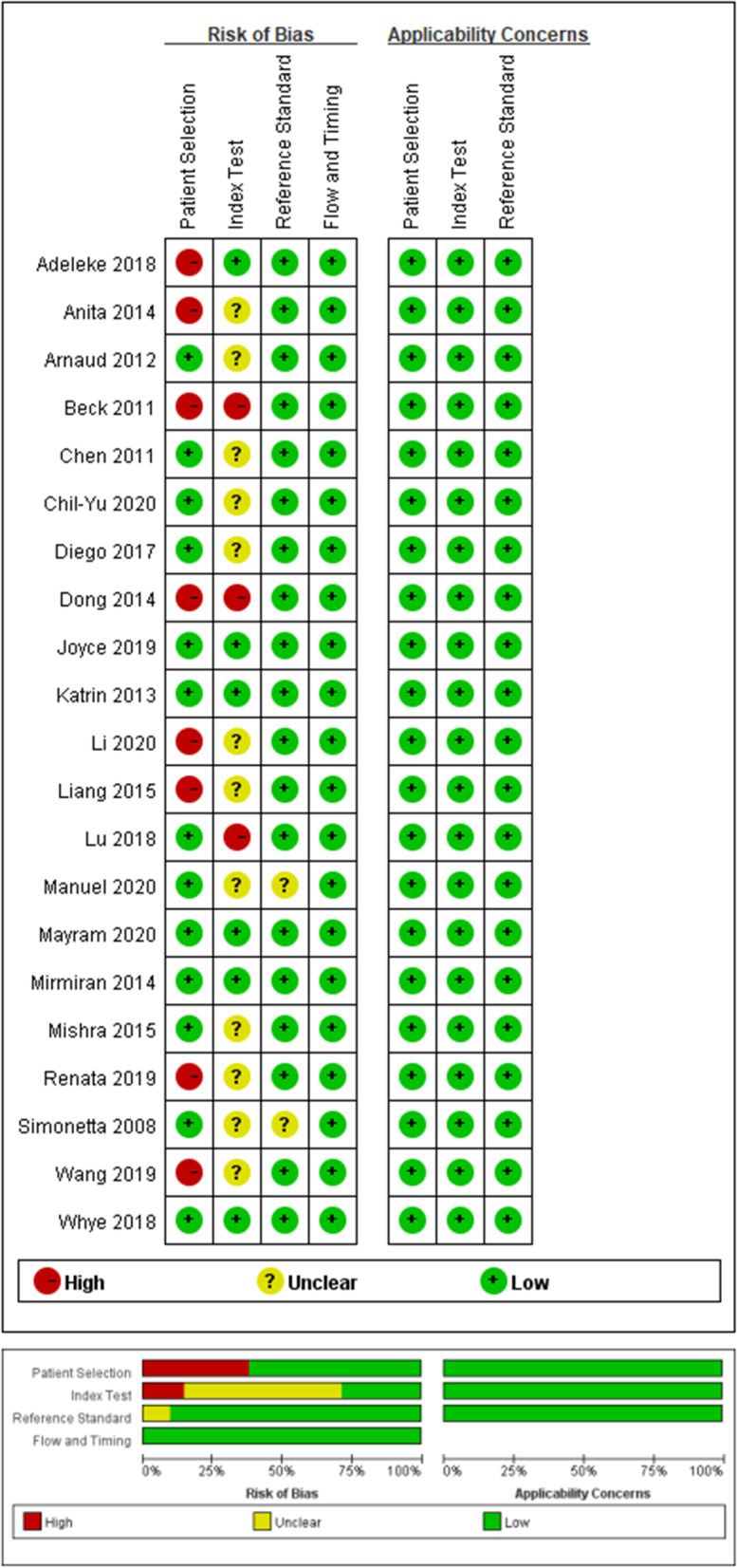
Methodological evaluation according to QUADAS-2 of included studies

Table 1 shows the characteristics of the 21 articles. Of the 21 articles, all of the studies reported BMI and WHtR, 20 studies reported WC. The total studies were cross-sectional and evaluated 177,943 children and adolescents 3–19 years of age. The detailed methodological quality assessment of included studies is shown in Fig. 2.

**Table 1:** Characteristics of included studies

Study	Study year	Country	Region	Study design	Chracteristics	BP measurement device	Sample number (boys/girls)	Age range (year) or mean (SD)	Hypertension criterion and basis	Anthropometric index
Simonetta [6]	2008	Italy	Europe	Cross-sectional	Hypertension	Mercury sphygmomanometer	4177(2172/2005)	5–11	≥95th percentile NHBPEP	BMI,WC,WHtR
Chen [21]	2011	China	Asia	Cross-sectional	Hypertension	Mercury sphygmomanometer	939(640/299)	3–6	≥ 95th percentile NHBPEP	BMI, WC, WHtR
Arnaud [22]	2012	Switzerland	Europe	Cross-sectional	Elevated blood pressure	Automated BP monitor	5207(2621/2586)	10–15	≥ 95th percentile NHBPEP	BMI, WHtR
Katrin [23]	2013	Germany	Europe	Cross-sectional	Elevated blood pressure	Automated BP monitor	6813(3492/3321)	11–17	≥95th percentile KiGGS	BMI,WHtR
Anita [24]	2014	Italy	Europe	Cross-sectional	Hypertension	Mercury sphygmomanometer	883(455/428)	8–18	≥95th percentile NHBPEP	BMI,WC,WHtR
Dong [25]	2015	China	Asia	Cross-sectional	Elevated blood pressure	Mercury sphygmomanometer	99,366(49,514/49852)	7–17	≥95th percentile NHBPEP	BMI,WC,WHtR
Beck [26]	2011	Brazil	America	Cross-sectional	Hypertension	Mercury sphygmomanometer	660 (343/317)	14–19	≥90th percentile Brazil	BMI,WC,WHtR
Liang [27]	2015	China	Asia	Cross-sectional	Elevated blood pressure	Mercury sphygmomanometer	5471(2799/2672)	6–10	≥95th percentile NHBPEP	BMI,WC,WHtR
Mirmiran [28]	2014	Iran	Asia	Cross-sectional	Hypertension	Mercury sphygmomanometer	134 (66/68)	10–18	≥90th percentile Iran	BMI,WC,WHtR
Mishra [29]	2015	India	Asia	Cross-sectional	Hypertension	Mercury sphygmomanometer	1913 (1111/802)	6–16	≥90th percentile NHBPEP	BMI,WC,WHtR
Diego [30]	2017	Brazil	America	Cross-sectional	Hypertension	Automated BP monitor	8295(4877/3418)	10–17	≥95th percentile NHBPEP	BMI,WC,WHtR
Adeleke [31]	2018	Canada	America	Cross-sectional	Hypertension	Automated BP monitor	762(360/402)	5.8–17	≥95th percentile NHBPEP	BMI,WC,WHtR
Lu [32]	2018	China	Asia	Cross-sectional	Elevated blood pressure	Mercury sphygmomanometer	1898(955/943)	7–15	≥ 95th percentile NHBPEP	BMI, WC, WHtR
Whye [33]	2018	Malaysia	Asia	Cross-sectional	Hypertension	Automated BP monitor	2461(1033/1428)	12–17	≥90th percentile Brazil	BMI,WC,WHtR
Joyce [34]	2019	Malaysia	Asia	Cross-sectional	Hypertension	Automated BP monitor	513(211/302)	12–16	≥95th percentile Malaysia	BMI,WC,WHtR
Renata [35]	2019	Lithuania	Europe	Cross-sectional	Hypertension	Automated BP monitor	7457(3494/3963)	12–15	≥95th percentile NHBPEP	BMI,WC,WHtR
Chih-Yu [36]	2020	China	Asia	Cross-sectional	Hypertension	Automated BP monitor	340(163/177)	7–12	≥95th percentile NHBPEP	BMI,WHtR
Wang [37]	2019	China	Asia	Cross-sectional	Hypertension	Mercury sphygmomanometer	683 (366/317)	8–15	≥95th percentile China	BMI,WC,WHtR
Li [38]	2020	China	Asia	Cross-sectional	Hypertension	Mercury sphygmomanometer	15,698(8004/7694)	6–17	≥95th percentile China	BMI,WC,WHtR
Manuel [39]	2020	Spain	Europe	Cross-sectional	Hypertension	Automated BP monitor	265(144/121)	6–16	≥95th percentile AEP	BMI,WC,WHtR
Mayram [40]	2020	Iran	Asia	Cross-sectional	Hypertension/elevated blood presure	Mercury sphygmomanometer	14,008(7091/6917)	7–18	≥90th; ≥95th percentile AAP	BMI,WC,WHtR

*BP* blood pressure, *BMI* body mass index, *WC* waist circumference, *WHtR* waist-to-height ratio

### Meta-analysis

**Fig. 3:**
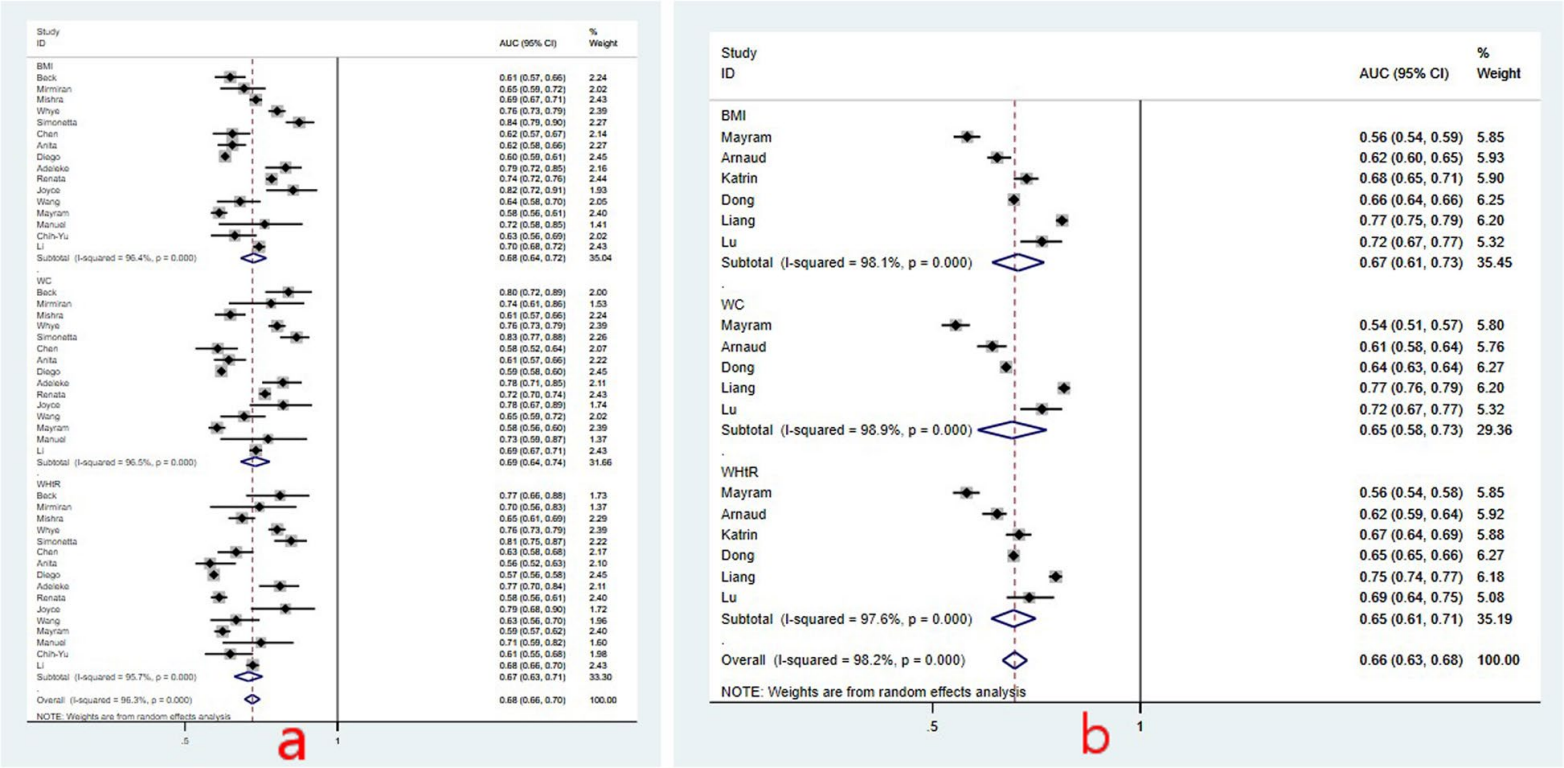
The pooled AUC values with 95%CI for hypertension and elevated BP in boys

**Fig. 4:**
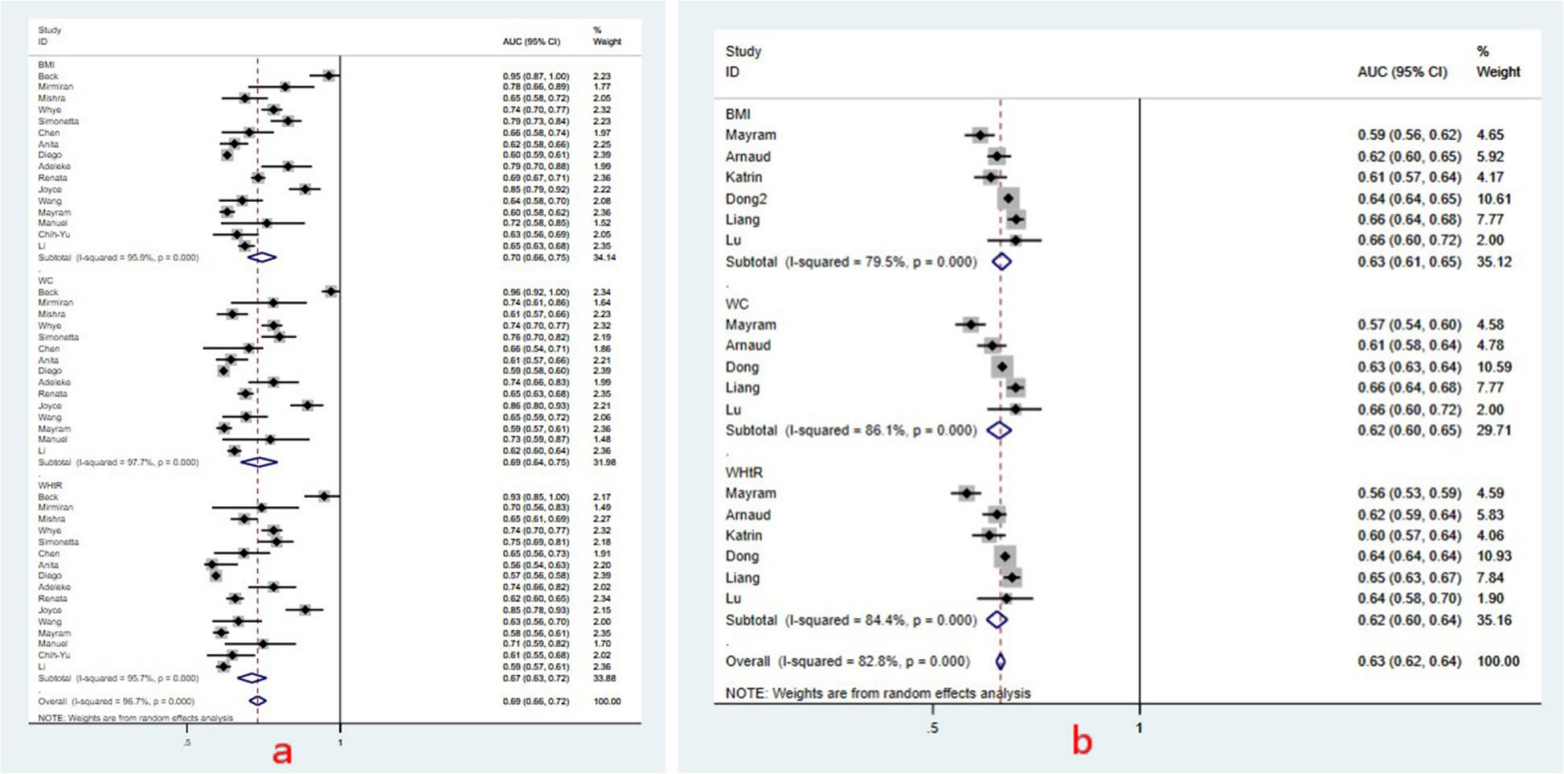
The pooled AUC values with 95%CI for hypertension and elevated BP in girls

The forest plots of the pooled area under the reporting receiver-operating characteristic curves (AUC) and 95% confidence intervals (CIs) for hypertension risk and elevated BP risk in boys were shown in Fig. 3. Figure 3a showed the AUC value of using BMI to predict hypertension risk was 0.68 (95%CI: 0.64, 0.72); the AUC values of WC and WHtR were 0.69 (95%CI: 0.64, 0.74) and 0.67 (95%CI: 0.63, 0.71); the heterogeneity across studies was *I*^2^ = 96.4%, *I*^2^ = 96.5%, and *I*^2^ = 95.7%. Figure 3b showed the pooled AUC values of using BMI, WC and WHtR to predict elevated BP risk were 0.67 (95%CI: 0.61, 0.73), 0.65 (95%CI: 0.58, 0.73) and 0.65 (95%CI: 0.61, 0.71) respectively; and the heterogeneity across studies was *I*^2^ = 98.1%, *I*^2^ = 98.9% and *I*^2^ = 97.6%, random-effects model was performed for meta-analysis. For girls, Fig. 4 showed the pooled AUC value for BMI, WC and WHtR of predicting hypertension risk and elevated BP risk. The pooled AUC values of using BMI, WC and WHtR to predict hypertension risk were shown in Fig. 4a, which were 0.70 (95%CI: 0.66, 0.75), 0.69 (95%CI: 0.64, 0.75) and 0.67 (95%CI: 0.63, 0.72), the heterogeneity across studies was *I*^2^ = 95.9%, *I*^2^ = 97.7% and *I*^2^ = 95.7%; Fig. 4b showed the pooled AUC values of using BMI, WC, and WHtR to predict elevated BP were 0.63 (95%CI: 0.61, 0.65), 0.62 (95%CI: 0.60, 0.65), 0.62 (95%CI: 0.60, 0.64) respectively, the heterogeneity across studies was *I*^2^ = 79.5%, *I*^2^ = 86.1% and *I*^2^ = 79.5%, similarly, random-effects model was used.

For the same anthropometric index, the ability of predicting hypertension and elevated BP was compared in the same gender (Figs. 3 and 4). The results showed that the AUC value of hypertension in children is higher than elevated BP, the accuracy of BMI in predicting hypertension is significantly higher than elevated BP in girls (*P* < 0.05), while the difference was not obvious in other indices (*P >* 0.05).

### GRADE classification

Regarding the quality of evidence in the study, since the design of included studies were observational studies, they were rated as low qualities at start, which had an impact on subsequent evidence ratings. It was classified as high quality by using BMI to predict hypertension risk in girls, and the result of using WHtR to predict hypertension risk in boys was rated as low quality (8%). GRADE guidelines classified other evidence as “moderate quality”, accounting for about 84%. The GRADE scores breakdown of each result were shown in Appendix 2.

### Subgroup analysis

Table 2 shows that the subgroup analysis of pooled AUC values and 95% CIs of BMI, WC, and the WHtR for hypertension and elevated BP risk. For regional analysis, blood pressure criterion (≥90th percentile and ≥ 95th percentile), diagnostic basis (NHBPEP and other local criteria of hypertension) and BP measurement device, there was no statistical significance existed between BMI, WC, and the WHtR for hypertension and elevated BP risk.

**Table 2:** Subgroup analysis of pooled AUC values with 95%CI in pediatric population

Subgroup (N of studies)	Hypertension				Subgroup (N of studies)	Elevated blood pressure			
	Boys		Girls			Boys		Girls	
	AUC(95%CI)	*I*^2^*(*%)	AUC(95%CI)	*I*^2^*(*%)		AUC(95%CI)	*I*^2^*(*%)	AUC(95%CI)	*I*^2^*(*%)
Region					Region				
Asia					Asia				
BMI (*n* = 9)	0.67 (0.63,0.72)	93.1	0.68 (0.63,0.74)	92.5	BMI (*n* = 4)	0.68 (0.60,0.76)	98.8	0.64 (0.62,0.67)	84.7
WC (*n* = 8)	0.67 (0.61,0.73)	94.1	0.68 (0.62,0.74)	94.3	WC (n = 4)	0.66 (0.58,0.76)	99.2	0.63 (0.60,0.66)	88.9
WHtR (n = 9)	0.67 (0.62,0.71)	91.4	0.66 (0.61,0.72)	93.6	WHtR (n = 4)	0.66 (0.60,0.73)	98.5	0.62 (0.59,0.65)	89.0
Europe					Europe				
BMI (n = 4)	0.73 (0.64,0.82)	93.1	0.70 (0.63,0.77)	90.9	BMI (*n* = 2)	0.65 (0.59,0.72)	91.0	0.62 (0.60,0.64)	0.0
WC (n = 4)	0.72 (0.64,0.81)	91.9	0.68 (0.62,0.74)	83.4	WC (n = 1)	0.61 (0.58,0.64)	–	0.61 (0.58,0.64)	–
WHtR (n = 4)	0.66 (0.55,0.79)	95.5	0.65 (0.58,0.73)	89.8	WHtR (n = 2)	0.64 (0.60,0.69)	83.0	0.61 (0.59,0.64)	0.0
America					America				
BMI (*n* = 3)	0.66 (0.57,0.76)	95.1	0.77 (0.55,1.00)	98.9	–	–	–	–	–
WC (n = 3)	0.71 (0.57,0.90)	96.9	0.75 (0.52,1.00)	99.4	–	–	–	–	–
WHtR (n = 3)	0.69 (0.54,0.88)	96.4	0.73 (0.52,1.00)	98.7	–	–	–	–	–
Hypertension criterion					Elevated blood pressure criterion				
≥90th percentile					≥90th percentile				
BMI (n = 4)	0.68 (0.63,0.74)	90.8	0.77 (0.66,0.91)	93.6	BMI (n = 1)	0.56 (0.54,0.59)	–	0.59 (0.56,0.62)	–
WC (n = 4)	0.72 (0.64,0.82)	89.9	0.75 (0.61,0.93)	91.3	WC (n = 1)	0.54 (0.52,0.57)	–	0.57 (0.54,0.60)	–
WHtR (n = 4)	0.72 (0.64,0.80)	84.4	0.75 (0.64,0.87)	93.6	WHtR (n = 1)	0.56 (0.54,0.59)	–	0.56 (0.53,0.59)	–
≥95th percentile					≥95th percentile				
BMI (*n* = 12)	0.68 (0.64,0.74)	96.9	0.68 (0.64,0.72)	94.5	BMI (*n* = 5)	0.69 (0.63,0.75)	97.9	0.64 (0.62,0.66)	65.1
WC (*n* = 11)	0.68 (0.63,0.73)	96.7	0.67 (0.63,0.71)	93.7	WC (n = 4)	0.68 (0.60,0.77)	99.0	0.64 (0.62,0.66)	74.8
WHtR (n = 12)	0.65 (0.61,0.70)	94.9	0.64 (0.61,0.68)	92.9	WHtR (n = 5)	0.68 (0.63,0.73)	97.3	0.63 (0.62,0.65)	49.1
Hypertension diagnosis basis					Elevated blood pressure diagnosis basis				
NHBPEP					NHBPEP				
BMI (n = 8)	0.69 (0.63,0.75)	97.6	0.67 (0.62,0.73)	94.1	BMI (n = 4)	0.69 (0.62,0.77)	98.5	0.65 (0.63,0.67)	68.7
WC (*n* = 7)	0.67 (0.60,0.74)	97.5	0.65 (0.61,0.70)	91.3	WC (n = 4)	0.68 (0.60,0.77)	99.0	0.64 (0.62,0.66)	74.8
WHtR (n = 8)	0.64 (0.59,0.70)	94.7	0.64 (0.59,0.68)	91.9	WHtR (n = 4)	0.68 (0.62,0.74)	98.0	0.64 (0.63,0.65)	32.0
Other local criteria of hypertension					Other local criteria of elevated blood pressure				
BMI (n = 8)	0.68 (0.62,0.74)	94.0	0.73 (0.65,0.82)	96.5	BMI (n = 2)	0.62 (0.51,0.75)	97.5	0.60 (0.57,0.62)	0.0
WC (n = 8)	0.71 (0.65,0.77)	93.8	0.73 (0.63,0.84)	98.2	WC (n = 1)	0.54 (0.52,0.57)	–	0.57 (0.54,0.60)	–
WHtR (n = 8)	0.70 (0.64,0.76)	92.3	0.71 (0.63,0.80)	96.6	WHtR (n = 2)	0.61 (0.51,0.73)	96.7	0.58 (0.54,0.63)	73.7
BP measurement device					BP measurement device				
Mercury sphygmomanometer					Mercury sphygmomanometer				
BMI (n = 9)	0.66 (0.62,0.71)	93.6	0.70 (0.63,0.77)	95.2	BMI (n = 4)	0.68 (0.60,0.76)	98.8	0.64 (0.62,0.67)	84.7
WC (n = 9)	0.67 (0.61,0.73)	93.8	0.68 (0.60,0.78)	97.9	WC (n = 4)	0.66 (0.58,0.76)	99.2	0.63 (0.60,0.66)	88.9
WHtR (n = 9)	0.66 (0.62,0.71)	90.3	0.66 (0.60,0.73)	94.5	WHtR (n = 4)	0.66 (0.60,0.73)	98.5	0.62 (0.59,0.65)	89.0
Automated BP monitor					Automated BP monitor				
BMI (n = 7)	0.72 (0.64,0.80)	97.9	0.71 (0.64,0.79)	96.8	BMI (n = 2)	0.65 (0.59,0.72)	91.0	0.62 (0.60,0.64)	0.0
WC (*n* = 6)	0.72 (0.64,0.81)	98.1	0.71 (0.63,0.80)	97.2	WC (n = 1)	0.61 (0.58,0.64)	–	0.61 (0.58,0.64)	–
WHtR (n = 7)	0.68 (0.60,0.76)	97.3	0.68 (0.61,0.77)	96.9	WHtR (n = 2)	0.64 (0.60,0.69)	83.0	0.61 (0.59,0.64)	0.0

*AUC* area under the curve, *CI* confidence interval, *BMI* body mass index, *WC* waist circumference, *WHtR* waist-to height ratio

### Publication bias and sensitivity analysis

Table 3 shows that no publication bias in hypertension and elevated BP were found according to the Begg’s test (hypertension-boys: *P* = 0.964, girls: *P* = 0.444 for BMI; boys: *P* = 0.692, girls: *P* = 0.198 for WC; boys: *P* = 0.558, girls: *P* = 0.260 for the WHtR; elevated BP-boys: *P* = 1.000, girls: *P* = 0.452 for BMI; boys: *P* = 0.462, girls: *P* = 0.806 for WC; boys: *P* = 1.000, girls: *P* = 0.260 for the WHtR). Sensitivity analysis was carried out and each study was excluded separately. The outcomes were not altered significantly when excluded. Indeed, none of the studies had a significant impact on the overall results.

**Table 3:** Results of publication bias

Anthropometric indices	N of studies	Gender	Begg’s *P*
BMI	16	boys	0.964
	16	girls	0.444
WC	15	boys	0.692
	15	girls	0.198
WHtR	16	boys	0.558
	16	girls	0.260
BMI	6	boys	1.000
	6	girls	0.452
WC	5	boys	0.462
	5	girls	0.806
WHtR	6	boys	1.000
	6	girls	0.260

## Discussion

This robust meta-analysis, which included 177,943 children and adolescents 3–19 years of age from populations all over the world, our studies comparison in the pediatric population showed that there is no statistical difference in the accuracy of BMI, WC, WHtR in screening children or adolescents for hypertension and elevated blood pressure. The evidence rating results of the GRADE guideline showed that most of the results were rated as moderate quality. The Chunming meta-analysis [12] of 9 cross-sectional studies included 25,424 children and adolescents 6–18 years of age in 7 different countries and confirmed that no anthropometric index was statistically superior to other anthropometric indices in identifying an elevated BP; on the basis of pooled AUC values, the AUC values were ranked in the following order, but this was not statistically significant: BMI (0.778) > WC (0.718) > WHtR (0.670). With the increased number of included articles in the current systematic review and increased number of subjects (*n* = 177,943), apparently, we obtained the same conclusion. Compare the ability of the same anthropometric index to predict hypertension risk and elevated BP risk in the same gender. The ability of BMI to predict hypertension risk was superior than predicting elevated BP risk, and this difference was extremely significant among girls. As a process of gradual abnormalities in BP, it is not enough to use anthropometric indices to predict elevated BP risk. Only when the BP changes from normal to hypertension and the change is more obvious, it seems to be more easier to make predictions.

According to the Chunming meta-analysis [12], the number of included studies was less, and which only included the studies based on NHBPEP as the diagnosis basis of hypertension. However, in terms of anthropometric index prediction, our study includes two characteristics of hypertension and elevated blood pressure, which means that we have analyzed both for the results of hypertension and the process of elevated blood pressure. Besides, the study not only included the studies based on NHBPEP as the diagnosis basis of hypertension, but also contained other local criteria of hypertension, because there is no clear and unified standard for the diagnosis of hypertension in children, some local hypertension diagnostic criteria are also applicable to local children and adolescents, which greatly improved the comprehensiveness of the included studies. In terms of subgroup analysis, we comprehensively consider the factors that may have an impact on results. One factor should be considered first was the regional issue, the prevalence of hypertension and elevated BP in different continents is different at present, and whether regional issue will affect the ability of the same index to predict hypertension risk in children, so we conducted the regional subgroup analysis. For the diagnostic basis of hypertension and elevated BP, partial included studies were based on the NHBPEP, and the rest were based on local diagnostic criteria. It seems to be more suitable to depend on the local diagnostic criteria to diagnose pediatric hypertension and elevated BP because it combines the BP and physical characteristics of local children. Therefore, the subgroup analysis for different diagnostic criteria of hypertension was performed. Similarly, the criterion of hypertension and elevated BP was P_90_ or P_95_ will also affect the ability of anthropometric indices to predict hypertension risk and elevated BP risk, and the measurement device may also affect the results. In view of these possible influencing factors, we also conducted subgroup analysis to increase the scientific nature of our study.

Both adults and children with overweight or obesity are associated with elevated BP [12, 42, 43] and weight loss can also improve BP levels in children with obesity [44, 45]. BMI, WC, and the WHtR are common indicators for predicting hypertension in children, the current meta-analysis suggested that the above three anthropometrics are all positively related to pediatric elevated blood pressure (AUC > 0.5). Among the anthropometric indices, BMI is most commonly used [46, 47] owing to its simplicity. However, the BMI also has shortcomings because BMI cannot distinguish between individuals with fattiness and individuals with muscle mass and cannot determine the location of fattiness [48, 49]. WC is often used to reflect abdominal obesity due to its strongly relationship with visceral fat depots [46]. It has been reported that body fat distribution has a close relationship to the occurrence and development of cardiovascular disease [50]. Visceral adipose tissue (VAT) accumulation, which is associated with an increase in free fatty acid content and insulin resistance, increases the risk of hypertension [51, 52]. WC and the WHtR are strongly related to abdominal obesity and VAT, which were assessed by radiologic examination [53, 54]. Therefore, a meta-analysis of adults showed that the WHtR is superior to WC and BMI in screening cardiovascular diseases [55], which confirmed that the index reflecting abdominal obesity is better than BMI in predicting metabolic risk. However, contrary to the research findings in adults, the analytical results in our review showed that no significant difference in BMI, WC and WHtR was found in predicting the risk of pediatric hypertension. This finding may reflect the rapid growth in stature from childhood to adolescence that outweighs the incremental change in WC, which greatly reduces the superiority of WHtR over WC and BMI to predict abdominal obesity [56]. However, our review showed that the ability of all the indicators to predict hypertension is higher than elevated blood pressure, especially BMI in girls. Limited by the number of included studies may lead to this result, another possible reason may be the early small increase in blood pressure in children is more secretive and difficult to find. Only when the blood pressure increases significantly and exceeds the critical value, can be easier to predict through anthropometric indicators. A previous study suggested that the WHtR decreases with age from 5 to 16 years until 18 years due to the cessation in growth [57]. That finding may also misclassify fast-growing children with excess abdominal fat as healthy. In addition, some studies involving children and adolescents showed that, unlike adults, BMI offers adequate information to evaluate visceral obesity and WC does not predict extra information [58]. Another study reported that the 95th BMI percentile of the Center for Disease Control is a useful threshold for predicting increased VAT, fattiness, and heart metabolic risk in children and adolescents [59].

ROC analysis has been a widely-used method for evaluating the efficacy of diagnostic tests. This meta-analysis focused on studies that used ROC method to compare the discriminatory power of anthropometric indices for elevated BP. However, there are still some limitations in this systematic review. First, the most important limitation is the different criteria of hypertension in the included studies. Most studies used NHBPEP criterion, and some studies applied local criterion. Therefore, both diagnostic standards were included and the subgroup analysis was conducted to explore the impact of anthropometric indicators predicting high BP and elevated BP. Some of them used the 90th percentile as a cutoff to diagnose hypertension and elevated BP, and some used the 95th percentile as a cutoff, so all the cutoff were included for subgroup analysis to explore further differences. Second, our study only considered three obesity anthropometric indices, yet other indices, such as the neck circumference [59], waist-to-hip ratio, and mid-upper arm circumference [60], were not used in this study because of the limited number of relevant papers. Finally, most results have the problem of high heterogeneity, though we have conducted the subgroup analysis to explore the possible sources of heterogeneity, the degree of heterogeneity did not decrease. Another regrettable issue was that for the timeliness of our study, PROSPERO was not registered in this review. In order to reduce bias, we can only make a protocol as required to assess the potential bias.

## Conclusion

In conclusion, this systematic review with a meta-analysis showed that no anthropometric index was statistically superior to other anthropometric indices in identifying hypertension and elevated BP in adolescents. However, for the same anthropometric index, the accuracy in predicting hypertension is better than elevated blood pressure, especially the application of BMI in girls. Further exploration is needed to find better anthropometric indicators for predicting early blood pressure abnormalities in children.

## Data Availability

The datasets generated and analysed during the current study are available from the corresponding author on reasonable request.

## Appendix 2 GRADE classification of quality of evidence

**Table:**

Outcome	Anthrop ometric	N of studies	Gender	Risk of bias	Inconsistency	Indirectness	Imprecision	Publication bias	Large effect	Dose response	Plausible confounding	Grade
	BMI	16	Boys	No serious	No serious	No serious	No serious	No serious	No	No	Would reduce effect	⨁⨁⨁○
		16	Girls	No serious	No serious	No serious	No serious	No serious	Large	No	Would reduce effect	⨁⨁⨁⨁
Hypertension	WC	15	Boys	No serious	No serious	No serious	No serious	No serious	No	No	Would reduce effect	⨁⨁⨁○
		15	Girls	No serious	No serious	No serious	No serious	No serious	No	No	Would reduce effect	⨁⨁⨁○
	WHtR	16	Boys	No serious	Serious	No serious	No serious	No serious	No	No	Would reduce effect	⨁⨁○○
		16	Girls	No serious	No serious	No serious	No serious	No serious	No	No	Would reduce effect	⨁⨁⨁○
	BMI	6	Boys	No serious	No serious	No serious	No serious	No serious	No	No	Would reduce effect	⨁⨁⨁○
Elevated BP		6	Girls	No serious	No serious	No serious	No serious	No serious	No	No	Would reduce effect	⨁⨁⨁○
	WC	5	Boys	No serious	No serious	No serious	No serious	No serious	No	No	Would reduce effect	⨁⨁⨁○
		5	Girls	No serious	No serious	No serious	No serious	No serious	No	No	Would reduce effect	⨁⨁⨁○

## Abbreviations

BP: Blood pressure
ROC: Receiver operating characteristics
BMI: Body mass index
WC: Waist circumference
WHtR: Waist-to-height ratio
AUC: Area under curve
SBP: Systolic blood pressure
DBP: Diastolic blood pressure
NHBPEP: National high blood pressure education program
QUADAS-2: Quality Assessment of Diagnostic Accuracy Studies-2
VAT: Visceral adipose tissue
